# Role of SUMO-1 and SUMO interacting motifs in rhesus TRIM5α-mediated restriction

**DOI:** 10.1186/1742-4690-10-10

**Published:** 2013-02-01

**Authors:** Zana Lukic, Stephen P Goff, Edward M Campbell, Gloria Arriagada

**Affiliations:** 1 Department of Microbiology and Immunology Stritch School of Medicine, Loyola University Chicago, 2160 S. 1st Ave. 508, Building 102, Room 5651, Maywood, IL, 60153, USA; 2 Department of Biochemistry and Molecular Biophysics, Columbia University, 701W168th street, HHSC1310, New York, NY 10032USA; 3 Howard Hughes Medical institute; 4 Departamento de Ciencias Biologicas, Facultad de Ciencias Biologicas, Universidad Andres Bello, Los Fresnos 52, Viña del Mar, Chile

**Keywords:** SUMO-1, SIM, rhTRIM5α, HIV-1

## Abstract

**Background:**

TRIM5α is a member of the tripartite motif family of proteins that restricts retroviral infection in a species-specific manner. The restriction requires an interaction between the viral capsid lattice and the B30.2/SPRY domain of TRIM5α. Previously, we determined that two SUMO interacting motifs (SIMs) present in the B30.2/SPRY domain of human TRIM5α (huTRIM5α) were important for the restriction of N-tropic Murine Leukemia Virus. Here, we examined whether SUMO expression and the SIM1 and SIM2 motifs in rhesus monkey TRIM5α (rhTRIM5α) are similarly important for Human Immunodeficiency Type 1 (HIV-) restriction.

**Results:**

We found that mutation of SIM1 and SIM2 of rhTRIM5α abolished the restriction of HIV-1 virus. Further, knockdown of SUMO-1 in rhTRIM5α expressing cells abolished restriction of HIV-1. These results may be due, in part, to the ability of SUMO-1 to stabilize rhTRIM5α protein expression, as SUMO-1 knockdown increased rhTRIM5α turnover and the mutations in SIM1 and SIM2 led to more rapid degradation than the wild type protein. The NF-κB signaling ability of rhTRIM5α was also attenuated by SUMO-1 knockdown. Finally, upon inhibition of CRM1-dependent nuclear export with Leptomycin B (LMB), wild type rhTRIM5α localized to SUMO-1 bodies in the nucleus, while the SIM1 and SIM2 mutants did not localize to SUMO-1.

**Conclusions:**

Our results suggest that the rhTRIM5α B30.2/SPRY domain is not only important for the recognition of the HIV-1 CA, but it is also important for its association with SUMO-1 or SUMO-1 modified proteins. These interactions help to maintain TRIM5α protein levels and its nuclear localization into specific nuclear bodies.

## Background

TRIM5α is a member of the TRIpartite Motif (TRIM) family of proteins, characterized as having three domains: a RING domain, either one or two B-box domains, and a coiled-coil domain (RBCC) [1]. TRIM5α is capable of restricting retroviral infection in a species-specific manner. This restriction requires an interaction between the retroviral capsid (CA) lattice and the B30.2/SPRY domain of TRIM5α [2-6]. The B30.2/SPRY domain, located at the C-terminal of TRIM5α, confers the restriction spectrum of TRIM5α proteins [6]. Human TRIM5α (huTRIM5α) potently restricts N-tropic murine leukemia virus (N-MLV) but it does not restrict B-tropic or NB-tropic MLV (B-MLV, NB-MLV respectively) [3,7]. On the other hand, rhesus macaque TRIM5α (rhTRIM5α) restricts N-MLV and human immunodeficiency type 1 (HIV-1) [6,8].

Small ubiquitin-related modifier (SUMO) proteins are conjugated to cellular substrates and regulate diverse cellular processes (for review see [9,10]). SUMO proteins are transferred to lysine residues within the UBC9 binding site of the target protein. This binding site has a consensus sequence ΨKXE (where Ψ is a hydrophobic residue, K is the lysine to which SUMO-1 is conjugated, X is any amino acid and E is glutamic acid) [11,12]. Conjugation of SUMO proteins to a substrate mediates distinct protein-protein interactions *in vivo*. These non-covalent interactions with SUMO modified proteins are mediated by SUMO interacting motifs (SIMs) [11,13,14]. The best-characterized SIMs have the consensus sequence V/I/L-x-V/I/L-V/I/L or V/I/L-V/I/L-x-V/I/L (where x is any amino acid) [14,15].

We have previously identified that SUMO conjugation system is involved in huTRIM5α- mediated restriction of N-MLV [16]. Three SIMs were identified in the huTRIM5α B30.2/SPRY domain. Two of them, SIM1 and SIM2, were responsible for the enhanced restriction of N-MLV in human cells observed upon SUMO-1 overexpression. Specifically, mutation of SIM1 and SIM2 resulted in a dramatic decrease in the ability of huTRIM5α to restrict N-MLV infection. The SIMs are conserved among TRIM5α orthologs, as the same SIM mutations also affected the rhTRIM5α restriction of N-MLV [16]. As such, we hypothesized that rhTRIM5α restriction of HIV-1 was similarly dependent on SUMO-1 expression and the SIM1 and SIM2 motifs of rhTRIM5α.

Here we report that SIMs are important for HIV-1 restriction, and that knockdown of SUMO-1 in rhTRIM5α expressing cells drastically reduces HIV-1 restriction. We hypothesize that the presence of SUMO-1 stabilizes proteins levels of rhTRIM5α, as knockdown of SUMO-1 decreases steady state levels of rhTRIM5α and NF-κB activation. Additionally, mutation of the SIMs abolishes the co-localization of rhTRIM5α with promyelocytic leukemia protein (PML, also known as TRIM19)/SUMO-1 in the nucleus upon LMB treatment. Our results suggest that the rhTRIM5α B30.2/SPRY domain is not only important for the recognition of the HIV-1 CA, but that it is also important for its association with SUMO-1 or with SUMO-1 modified proteins. This association helps maintain TRIM5α protein levels, as well as its ability to mediate NF-κB, and nuclear localization into specific nuclear bodies.

## Results and discussion

### Mutations in rhTRIM5α SIM1 and SIM2 motifs abolish HIV-1 restriction

**Figure 1:**
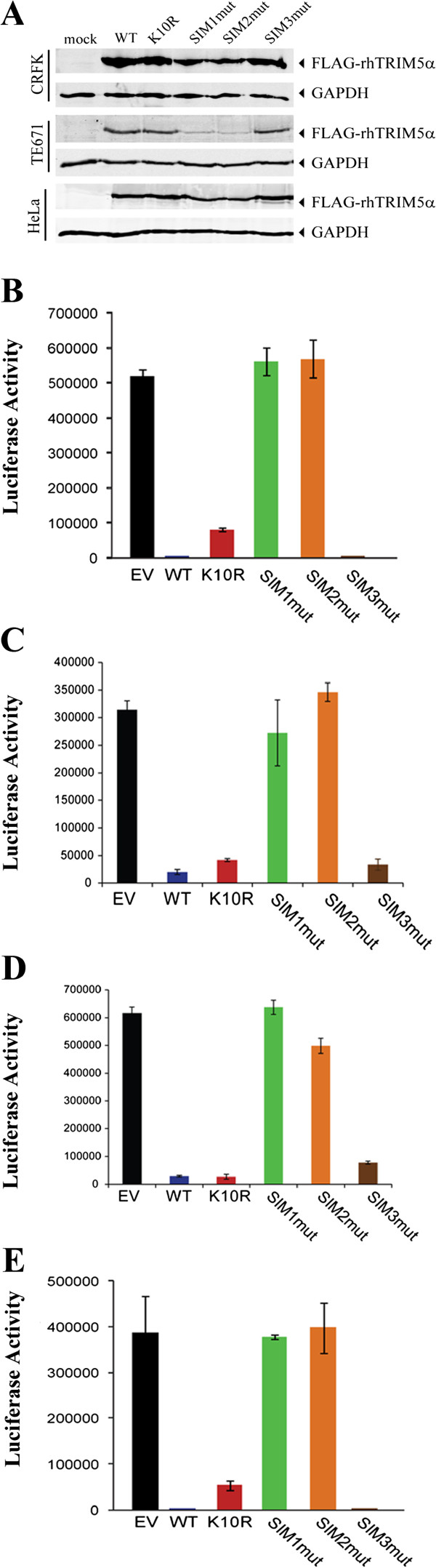
**SUMO interacting motifs are important for HIV-1 restriction by rhesus macaque TRIM5α.** CRFK, TE671 and HeLa cells were transduced with a retroviral vector encoding wild type rhTRIM5α, SIM1mut, SIM2mut and SIM3mut or an empty vector (EV) control. **A**. Western blot assay showing the expression of the FLAG-tagged rhTRIM5α, using an anti-FLAG antibody, GAPDH was used as a loading control. **B**-**C**. Cells stably expressing the various rhTRIM5α proteins or an EV control were infected with an HIV-1 firefly luciferase reporter virus pseudotyped with VSV-G envelope. 48 hours after infection luciferase activity was measured. Representative experiment of four independent experiments for CRFK (**B**), HeLa (**C**) and TE671 (**D**) cells. **E**. The CRFK cells were infected with an N-MLV firefly luciferase reporter virus pseudotyped with VSV-G envelope. Representative experiment of four independent experiments. Error bars show standard deviation between triplicates.

To explore the importance of rhTRIM5α SIMs in restriction of HIV-1, we generated CRFK, HeLa, and TE671 cell lines stably expressing comparable levels of FLAG-tagged wild type rhTRIM5α or the rhTRIM5α variants with mutations in SIM1 (376–379), SIM2 (405–408) and SIM3 (430–433) (Figure 1A). These cell lines were infected with VSV-G pseudotyped HIV-1 carrying a firefly luciferase reporter gene to assess retroviral restriction. The wild type and SIM3 rhTRIM5α efficiently restricted HIV-1 infection when compared to the empty vector (EV) in CRKF (Figure 1B) and HeLa cells (Figure 1C). Conversely, mutation of SIM1 and SIM2 of rhTRIM5α completely abolished the restriction activity in these cell lines (Figure 1B, C). Similar results were observed in TE671 cells (Figure 1D), although the expression of the SIM1 and SIM2 mutants was noticeably reduced compared to the expression of wild type rhTRIM5α (Figure 1A). In all cell lines, mutation of rhTRIM5α lysine 10 to arginine (K10R), a predicted SUMOylation site had minimal effect on restriction, consistent with our previous observations of huTRIM5α and rhTRIM5α N-MLV restriction [16]. Similarly, in CRFK cells, which do not express a functional TRIM5 gene [17], the restriction of N-MLV by rhTRIM5α required a functional SIM1 and SIM2, recapitulating the restriction profile observed for HIV-1 (Figure 1E). Therefore, SIM1 and SIM2 present in rhTRIM5α are important for its antiviral activity against both N-MLV and HIV-1. Consistent with this observation, another group has recently reported that the SIM1 and SIM2 mutations disrupt the binding of rhTRIM5α to the HIV-1 capsid [18].

### Restriction of HIV-1 by rhTRIM5α is reduced following SUMO-1 knockdown

**Figure 2:**
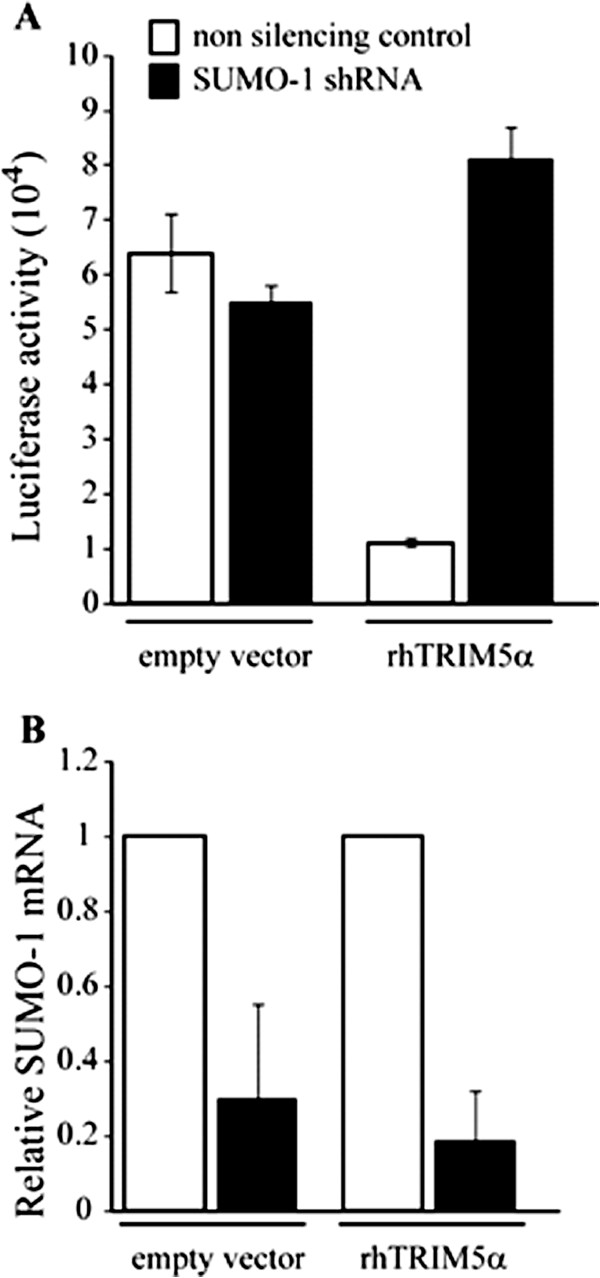
**Restriction of HIV-1 by rhTRIM5α is reduced following SUMO-1 knockdown.** The TE671 empty vector control cell line or the FLAG-rh TRIM5α expressing cell line were transduced with a retroviral vector encoding a non-silencing shRNA (white bars), or a SUMO-1 specific shRNA (black bars) and selected by antibiotic resistance. **A**. The different cell lines were infected with an HIV-1 firefly luciferase reporter virus pseudotyped with VSV-G envelope. 48 hours post infection, luciferase activity was measured. Representative experiment of three independent experiments. Error bars show standard deviation between triplicates. **B**. RNA was extracted, and the mRNA levels of SUMO-1 were determined by quantitative PCR. The values were normalized to GAPDH mRNA and expressed as fold over the respective non-silencing control. Error bars indicate standard deviation between three different quantifications**.**

To determine if the ability of rhTRIM5α to restrict HIV-1 infection was dependent on interactions with SUMO-1, we stably knocked down SUMO-1 (SUMO-1 KD) using a SUMO-1 specific shRNA in TE671 cells expressing FLAG-rhTRIM5α or empty vector. A non-silencing shRNA was used as a control. To confirm SUMO-1 KD, we performed quantitative PCR (qPCR) and found that these cells had ~70% SUMO-1 KD (Figure 2B). Control cells transduced with empty vector did not show appreciable differences in HIV-1 infection following SUMO-1 KD (Figure 2A). Cells transduced to express FLAG-rhTRIM5α showed considerable restriction of HIV-1. Notably, following SUMO-1 KD the restriction activity of cells expressing FLAG-rhTRIM5α was reduced to levels similar to cells expressing empty vector (Figure 2A). This demonstrates that rhTRIM5α restriction of HIV-1 is sensitive to SUMO-1 depletion.

### SUMO-1 enhances rhTRIM5α stability in cells

**Figure 3:**
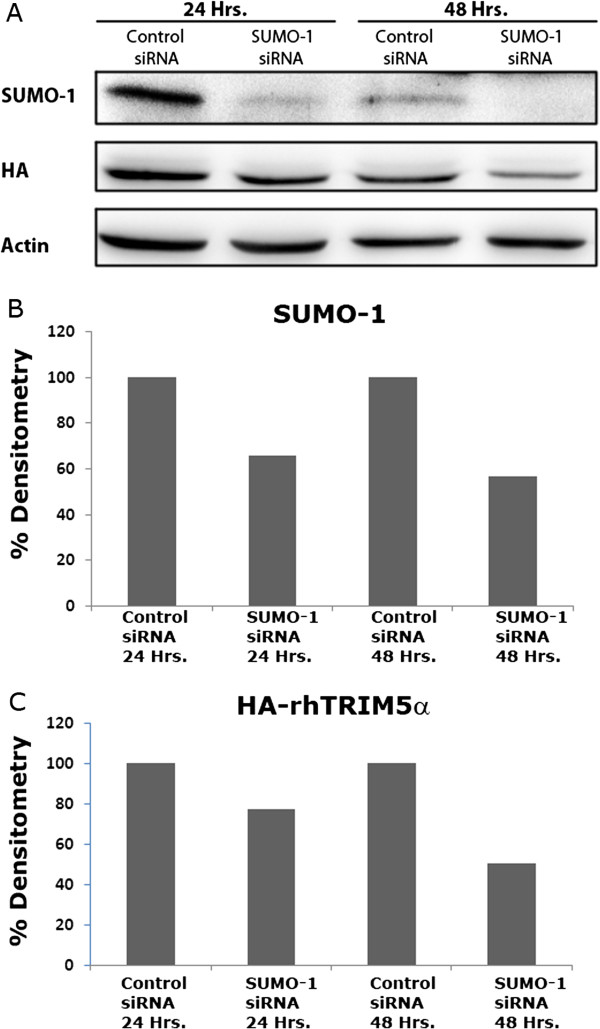
**SUMO-1 enhances rhTRIM5α stability in cells. A**. HeLa cells stably expressing HA-rhTRIM5α were transfected with siRNA targeting SUMO-1 or a non-targeting siRNA. Cells were rested for 24 or 48 hours post transfection. Cell lysates were collected and analyzed by Western blot for SUMO-1, HA-rhTRIM5α and β-actin. Representative Western blot image of 5 independent experiments. **B**. Densitometry analysis using ImageJ on SUMO-1 protein levels. SUMO-1 densitometry in SUMO-1 siRNA treated cells was normalized to control siRNA treated cells. **C**. HA-rhTRIM5α densitometry analysis using ImageJ on HA-rhTRIM5α protein levels. Densitometry was normalized as in B.

As noted earlier, TE671 cells expressing rhTRIM5α SIM mutants showed reduced expression compared to TE671 cell lines expressing wild type or K10R forms of rhTRIM5α (Figure 1A). This suggests that disrupting interactions with SUMO-1 may increase the turnover of rhTRIM5α. It was previously reported that knockdown of host cellular proteins which interact with rhTRIM5α increased rhTRIM5α turnover [19]. We therefore examined rhTRIM5α expression levels by Western blot following SUMO-1 siRNA treatment in a HeLa cell line stably expressing HA-rhTRIM5α [8]. In these cells, SUMO-1 KD reduced HA-rhTRIM5α expression (Figure 3), although this reduction did not correlate with the degree of restriction observed (Figure 2B). Other studies have noted that small alterations in TRIM5α expression do not dramatically affect restriction activity at non-saturating amounts of virus [4,20]. These observations make it difficult to separate the contribution of reduced protein expression and the relief of restriction observed in studies of this type. Therefore, it remains possible that the effects of SUMO-1 KD on restriction are not entirely due to reduced expression of rhTRIM5α.

### NF-κB activation by rhTRIM5α is sensitive to SUMO-1 expression

**Figure 4:**
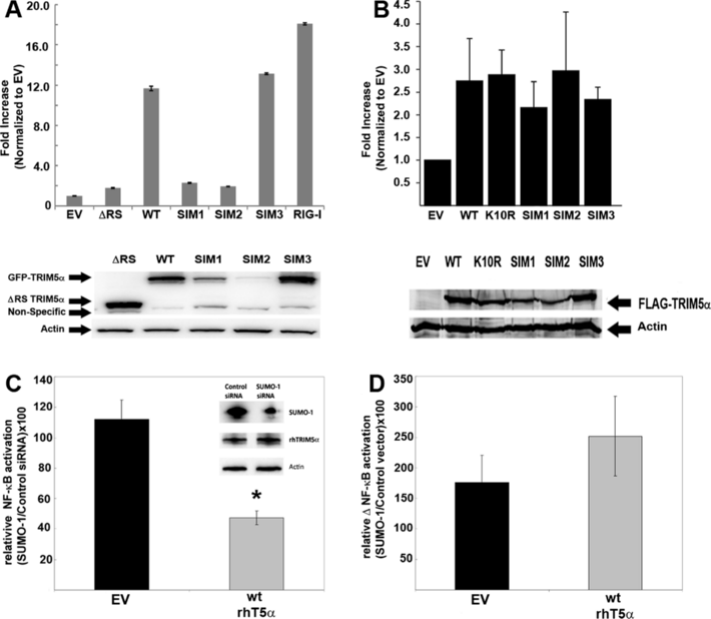
**The role of SIMs and SUMO-1 expression on NF-κB activation by rhTRIM5α. A**. 293T cells plated in triplicate were transfected with empty vector (EV), ΔRING/SPRY rhTRIM5α (ΔRS), wild typerhTRIM5α, SIM mutants or RIG-I along with NF-κB-responsive firefly luciferase construct. A renila luciferase construct was used as an internal transfection efficiency control. 48-hours post transfection, cells were lysed and luciferase activity was measured. NF-κB luciferase readings were normalized to renilla luciferase, and plotted as an average fold increase over empty vector. Upper panel, representative luciferase activity in the presence of various constructs.Error bars represent the SEM between the triplicates. Lower panel,representative Western blot. Representative of 4 independent experiments. **B**. 293A stably expressing wild typerhTRIM5α, K10R mut, SIM mutants with a FLAG-tag or an EV. Cells weretransiently transfected with an NF-κB firefly luciferase and renila luciferase plasmids as in A. Upper panel, NF-κB luciferase activity in the presence of rhTRIM5α mutants. Error bars represent the SD between 3 independent experiments. Lower panel, representative Western blot at the time of data acquisition. **C**. 293T cells were transfected with Control siRNA or SUMO-1siRNA for 48 hours. Cells were then seeded in a 96-well plate in triplicate and transfected with empty vector or wild type rhTRIM5α. NF-κB activity was measured as in A. Data were plotted by dividing SUMO-1 siRNA activation by control siRNA activation x 100. Inset, representative Western blot. P <0.004 by Student’s t-test. Error bars represent the SEM between the triplicates. Data is representative of 3independent experiments. **D**. 293T cells were transfected with empty vector, and wild type rhTRIM5α constructs in presence and absence of SUMO-1. NF-κB activity was measured as in A. Data were plotted as in C. Error bars represent the SEM between triplicates. Representative of 3 independent experiments.

Recent studies have shown that TRIM5 proteins can activate intracellular signaling pathways that culminate in AP-1 and NF-κB activation [21,22]. In order to understand the role of SUMO-1 and SIMs in rhTRIM5α-mediated signaling, we transiently expressed wild type and rhTRIM5α mutants along with an NF-κB luciferase reporter. Both the wild type and SIM3 mutant form of rhTRIM5α were able to activate NF-κB. On the other hand, the SIM1 and SIM2 mutants did not induce significant signaling above background in this context (Figure 4A, top panel). However, following transient transfection, the protein expression levels of the SIM1 and SIM2 mutants were reduced compared to wild type and SIM3 mutants, possibly explaining the loss of NF-κB activation (Figure 4A, bottom panel). To assess NF-κB signaling by rhTRIM5α SIM mutants at comparable protein levels, we generated 293A cell lines stably expressing wild type rhTRIM5α and the SIM mutants and measured NF-κB activation in these cells. Under these conditions, when the SIM mutants were expressed at comparable levels to wild type rhTRIM5α, they elicited similar levels of NF-κB activation (Figure 4B). Consistent with a previous report that showed normal oligomerization of these mutants [18], and the data here that demonstrate the ability of these mutants to activate NF-κB, we conclude that the defect in restriction by rhTRIM5α SIM mutants is not due to gross misfolding of the protein.

We next asked how SUMO-1 depletion affected the ability of wild type rhTRIM5α to induce NF-κB activation. We co-transfected 293T cells with wild type HA-rhTRIM5α, SUMO-1 or control siRNA and an NF-κB driven luciferase reporter. We measured NF-κB activation by rhTRIM5α in SUMO-1 siRNA treated cells compared to rhTRIM5α cells treated with control siRNA. As shown in Figure 4C knocking down SUMO-1 had little effect on NF-κB activation when transfected with EV (~10%, black bar). However, depletion of SUMO-1 significantly reduced (~60%, p<0.004, Student’s T-test) NF-κB activation by wild type rhTRIM5α (Figure 4C, grey bar). The reduction of NF-κB activation in the presence of SUMO-1 siRNA was not due to reduced rhTRIM5α protein levels (Figure 4C inset) as SUMO-1 knockdown does in cells stably expressing rhTRIM5α (Figure 3). Conversely, overexpression of SUMO-1 increased NF-κB activation following transfection with empty vector or vector expressing wild type rhTRIM5α (Figure 4D). This increase was more pronounced when rhTRIM5α was present, although this result was not statistically significant (p=0.187, Student T-test). These experiments demonstrate that the ability to associate with SUMO-1 or SUMOylated proteins is relevant to rhTRIM5α-mediated NF-κB signaling**.**

### The SIM1 and SIM2 mutations disrupt rhTRIM5α trafficking to nuclear bodies containing PML and SUMO-1

**Figure 5:**
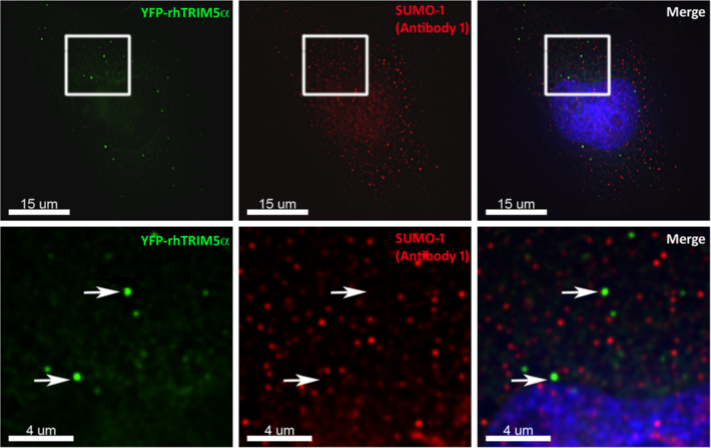
**SUMO-1 antibody recognizing cytoplasmic SUMO-1 does not localize to rhTRIM5α cytoplasmic bodies.** HeLa cells stably expressing YFP-rhTRIM5α were immunostained with a mouse anti-SUMO-1 (GMP1) clone 21C7. The white box in the top panel represents the area that was zoomed in to create the bottom panel. White arrows point to representative rhTRIM5α cytoplasmic bodies that do not contain SUMO-1.

**Figure 6:**
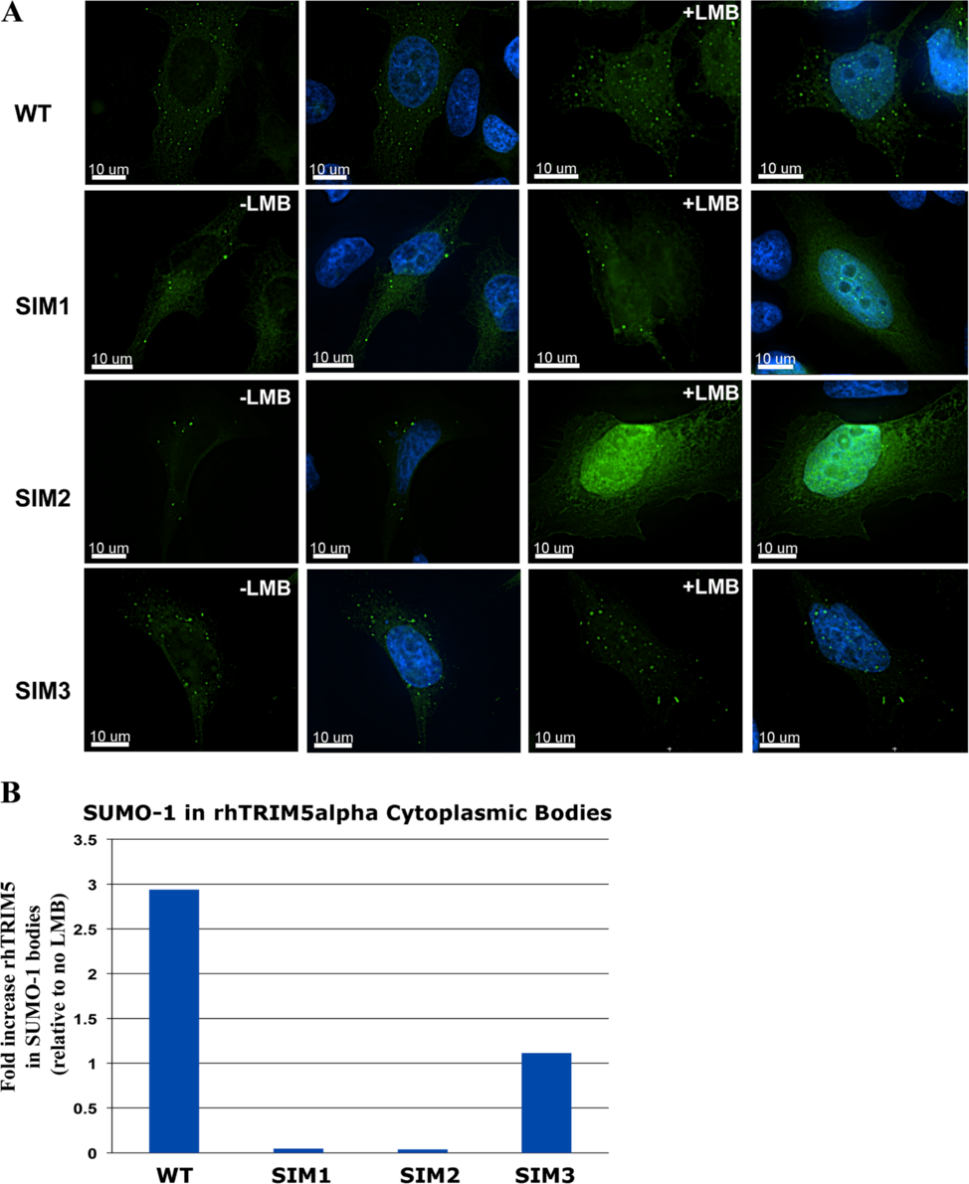
**The SIM1 and SIM2 mutations disrupt rhTRIM5α localization to nuclear bodies containing PML/TRIM19 andSUMO-1. A**. HeLa cells stably expressing wild typeYFP-rhTRIM5α or SIM mutants were treated with LMB for 4 hours. Following treatment the cells were fixed and imaged. Z-stack images were collected with a DeltaVision microscope equipped with a digital camera using a 1.4-numerical aperture (NA) 100× objective lens, and were deconvolved with SoftWoRx deconvolution software. Individual channel images were superimposed to create the merged panels. **B**. HeLa cells expressing wild type YFP-rhTRIM5a or SIM mutants were treated with LMB for 4hours. Following treatment the cells were fixed and stained with an anti-SUMO-1 antibody. Z-stack images were collected as described in A. Deconvolved images were analyzed for YFP-rhTRIM5a maximum fluorescence intensity (MFI) in SUMO-1nuclear bodies by the use of the Surface Finder function in the Imaris software(Bitplane). For each SUMO-1 puncta, the MFI of YFP-rhTRIM5a mutants was determined and the data was plotted in GraphPad Prism 5® software.

We next analyzed the association of rhTRIM5α and SUMO-1 by immunofluorescence to verify that SIM1 and SIM2 do not interact with SUMO-1 or SUMO-1 modified proteins. In HeLa cells stably expressing YFP-rhTRIM5α, we examined the co-localization of rhTRIM5α and endogenous SUMO-1. We used two antibodies to SUMO-1 to examine both the cytoplasmic and nuclear fractions of SUMO-1. The first antibody (GMP1, clone 21C7) recognized nuclear SUMO-1 as well as numerous cytoplasmic puncta. However, the cytoplasmic SUMO-1 did not co-localize with YFP-rhTRIM5α (Figure 5). When these cells were stained with an antibody to PML, we did not observe significant localization of SUMO-1 to PML (data not shown). As SUMO-1 positive structures in the nucleus are well characterized and known to contain PML [23,24], we used a second antibody (clone Y299) that recognized nuclear structures that were PML positive (Data not shown). This antibody detected primarily diffuse and punctate nuclear SUMO-1. We used this antibody in subsequent experiments to examine SIM1 and SIM2 localization with SUMO-1. A recent study demonstrated that while steady state rhTRIM5α is excluded from the nucleus, it can transiently enter and exits the nucleus, where it associates with PML bodies. This nuclear localization of rhTRIM5α to PML bodies is observed when the nuclear export of rhTRIM5α is inhibited with Leptomycin B (LMB), which is an inhibitor of CRM1 mediated nuclear export [25]. Inhibiting the nuclear export of rhTRIM5α using LMB revealed that wt and all three SIM mutants localized to the nucleus (Figure 6A), contrary to a recent report by another group which found that the SIM1 and SIM2 mutants did not localize to the nucleus under these conditions [18]. However, when we quantified the localization of these mutants to nuclear SUMO-1 bodies, SIM1 and SIM2 mutants of rhTRIM5α failed to localize to SUMO-1 positive bodies when nuclear export is inhibited, while the SIM3 mutant associated with these bodies to an intermediate degree (Figure 6B). These SUMO-1 positive rhTRIM5α nuclear bodies were also positive for PML (data not shown).

### Potential mechanisms of SUMO-1 regulation of rhTRIM5α

Collectively, the data presented here demonstrate that SIMs in rhTRIM5α and SUMO-1 are important for rhTRIM5α-mediated retroviral restriction. rhTRIM5α is sensitive to changes in SUMO-1 expression as SUMO-1 knockdown relieves rhTRIM5α restriction (Figure 2) and increases rhTRIM5α protein turnover in stable cells (Figure 3). Additionally, SUMO-1 modulates rhTRIM5α-dependent activation of NF-κB (Figure 4). Upon SUMO-1 knockdown, NF-κB activation is decreased in a rhTRIM5α-dependent manner (Figure 4C). We also observed decreased localization of SIM1 and SIM2 to SUMO-1 puncta following LMB treatment (Figure 6).

The mechanism by which SUMO-1 stabilizes rhTRIM5α expression or affects retroviral restriction is unclear. It is possible that rhTRIM5α interacts with a protein that stabilizes its expression in a SUMO-dependent manner. It is also possible that the expression of another cellular protein that is stabilized by SUMOylation, stabilizes rhTRIM5α expression. Consistent with this, it is known that SUMOylation can protect some proteins from ubiquitin dependent degradation [26]. Our observation that the SIM1 and SIM2 mutations, which abrogate restriction, are also turned over more rapidly than wild type rhTRIM5α are consistent with all of these possibilities. Interestingly, a recent study by Brandariz-Nunez *et al*. suggests that these residues are not present on the surface of the SPRY domain [18], as modeled using an NMR structure recently described for the PRY/SPRY domain [27]. It may be that conformational changes induced by the binding of other cellular factors conditionally expose the SIM1 and SIM2 motifs. These authors also observe that SIM1 and SIM2 mutants oligomerize normally [18], suggesting these proteins are not misfolded. While we cannot exclude the possibility that SIM1 and SIM2 mutations prevent proper folding of the B30.2/SPRY domain, our own results demonstrate that alterations in SUMO-1 expression can influence the stability of wild type rhTRIM5α.

The data presented here may begin to explain the mechanistic of rhTRIM5α nuclear trafficking. It is possible that the recruitment of TRIM5α to SUMO-1/PML bodies has direct relevance to the restriction process, as both the SIM1 and SIM2 mutants that do not localize to SUMO-1/PML bodies are completely unable to restrict retroviral infection. Considering that PML protein undergoes SUMOylation on three lysine residues [28-30] and rhTRIM5α has a rapid turnover [31], we can speculate that the steady state levels of rhTRIM5α protein are maintained by its interaction via SIMs with SUMOylated-PML in the nucleus. When SIMs or SUMO-1 are not present rhTRIM5α is less retained in the nucleus and therefore is degraded faster in the cytosol, reducing the amount of protein available to restrict incoming viruses.

Interestingly, owl monkey TRIM-Cyp, which contains a C-terminal cyclophilin A domain instead of a B302/SPRY domain (the B30.2/SPRY domain contains all three putative SIMs) [32], does not traffic to the nucleus [25]. Therefore, given the strong homology between the RBCC domains of owl monkey TRIM-Cyp and rhTRIM5α, it is possible that the SPRY domain contains the determinants that govern nuclear trafficking. Consistent with this hypothesis, our data demonstrate that wild type rhTRIM5α, but not the SIM1 and SIM2 mutants, localizes to nuclear SUMO-1 bodies (Figure 5) support this idea. Although the results we have obtained using the SIM1 and SIM2 mutants were consistent with the effect of SUMO-1 knockdown on wild type rhTRIM5α activity, the results obtained with these mutants should be cautiously interpreted [18].

Future studies are needed to determine if this association is merely correlative or the association of rhTRIM5α with nuclear SUMO-1/PML bodies potentiates specific steps in the restriction process.

## Conclusions

Our results demonstrate that rhTRIM5α restriction and expression is governed by SUMO-1 expression. SUMO-1 depletion, or mutations that are expected to disrupt SUMO-1 binding, abrogate restriction and increase the rate of rhTRIM5α turnover. The ability of rhTRIM5α to activate innate immune signaling pathways is also sensitive to SUMO-1 expression. Additionally, the ability to traffic to nuclear SUMO-1 bodies is abrogated by mutation of the SIM1 or SIM2 motifs present in rhTRIM5α. As rhTRIM5α does not appear to itself be SUMOylated, future studies are needed to identify the cellular factor or factors that are likely regulating rhTRIM5α in a SUMO-1 dependent fashion.

## Methods

### Cell lines and plasmids

Human embryonic fibroblast 293T, 293A, human medulloblastoma cell line TE671, HeLa and Crandall feline kidney (CRFK) fibroblast were maintained in Dulbecco’s modified Eagle medium supplemented with 10% fetal bovine serum, 100 UI/ml penicillin and 100 mg/ml streptomycin. All cells were cultured at 37°C in 5% CO_2_.

The rhTRIM5α constructs encoding the FLAG tagged wild type and mutant versions were identified and cloned as previously described [16]. YFP tagged wild type and mutant versions were cloned into a retroviral vector previously described [33]. RIG-I construct was kindly donated to us by Dr. Susan Baker (Loyola University Chicago).

### Generation of stable cell lines

Retroviruses for transduction were produced by transfection of 293T cells with 1 μg pMD.G, 1 μg pCMVI and 1.5 μg of either pQCXIN, pQCXIN-FLAG-rhTRIM5α wild-type or mutant versions, using FUGENE (Roche). Viruses were harvested 48 h after transfection, filtered (0.45 μm) and used to infect 5x10^5^ cells in 100 mm dishes in the presence of 8 μg/ml polybrene. HeLa, TE671, 293A cells infected with vectors delivering the Neo^r^ gene were selected in 1.5 μg/ml G418. The CRFK cells overexpressing rhTRIM5α were previously described [16]. Lentiviruses for transduction were produced by transfection of 293T cells with 1 μg pMD.G, 1 μg p8.91 and 1.5 μg of pGIPz (Open Biosystems) or pGIPzSUMO-1 DNAs containing shRNA (Open Biosystems). Viruses were harvested 48 h after infection, filtered (0.45 μm) and used to infect 5x10^4^ TE671-rhTRIM5α cells in 35 mm dishes in the presence of 8 μg/ml polybrene. Cells were selected in 1.5 μg/ml G418 and 1.5 μg/ml puromycin.

### Western blotting

Cells were lysed in 20 mMTris-HCl (pH 8.0), 137 mMKCl, 10% glycerol, 1% NP-40 and complete protease inhibitor (Roche) or Reporter lysis buffer (Promega). Samples were then boiled in 5x sodium dodecyl sulphate (SDS) loading buffer, and the proteins were resolved by SDS-polyacrylamide gel electrophoresis (PAGE). After transfer to nitrocellulose membranes, the blots were probed with mouse anti-β actin (Sigma), anti-HA (Covance), anti SUMO-1 (Abcam clone Y299), anti-FLAG (Sigma) or anti-GAPDH (Calbiochem).

### Single-cycle infectivity assay

HIV-1 luciferase reporter virus was produced by transfection of 293T cells with 2μg pNL4.3-env luciferase, and 1 μg pMD.G (per 100 mm plate) using FUGENE (Roche). Reporter virus stocks were harvested 48 h after transfection, then filtered (0.45 μm) and stored at −80°C. 293A (3x10^4^ per well), HeLa (2.5x10^4^ per well), TE671 (2.5x10^4^ per well) and CRFK (3x10^4^ per well) cells were seeded in 24-well plates and infected with MLV luc reporter viruses. Forty-eight hours post-infection cells were collected and assayed for firefly luciferase activity (Promega) in a luminometer.

### Analysis of SUMO-1 knock down

In the case of the TE671 cells where SUMO-1 was stably knocked down, the cells were harvested and total RNA was extracted using TRIZOL reagent (Invitrogen). 2 μg of total RNA per cell line were used to produce cDNA using random hexamers and SuperScript III kit (Invitrogen). 2 μl of each cDNA were used for quantitative PCR analysis of SUMO-1 and GAPDH transcript levels. Fold change was calculated using the relative standard curve method.

For the transient knockdown of SUMO-1, HeLa cells stably expressing HA-rhTRIM5α were transfected with siRNA specifically targeting SUMO-1 or a non-targeting siRNA (Santa Cruz Biotechnology). Cells were transfected with Lipofectamine2000 (Invitrogen) using a 2-day transfection protocol followed by 24-hours or 48-hours of rest to gain maximum knockdown efficiency. Cells were collected following the resting period and analyzed by western blot using a monoclonal anti-SUMO-1 antibody (Abcam clone Y299). Densitometry analysis was performed on the western blots using ImageJ software.

### Immunofluorescence

HeLa cells transfected with wild type YFP-rhTRIM5α and SIM mutants (Lipofectamine2000 protocol) were allowed to adhere to fibronectin-treated glass coverslips. Cells were treated with LMB for 4 hours and fixed with 3.7% formaldehyde (Polysciences) in 0.1 M PIPES, pH 6.8 [piperazine-*N*, *N*'-bis(2-ethanesulfonic acid)] (Sigma). Monoclonal rabbit anti-SUMO-1 (Abcam clone Y299) antibody was used to stain SUMO-1. Primary antibody was secondarily labeled with Cy5 fluorophore-conjugated donkey anti-mouse antibody (Jackson ImmunoResearch). Nucleus was stained using a DAPI stain (Jackson ImmunoResearch). Images were collected with a DeltaVision microscope (Applied Precision) equipped with a digital camera (CoolSNAP HQ; Photometrics), using a 1.4-numerical aperture 100× objective lens, and they were deconvolved with SoftWoRx deconvolution software (Applied Precision).

### Image analysis

20 Z-stack images were acquired using identical acquisition parameters. Surfaces for cytoplasmic bodies in all samples analyzed were defined by using a fluorescence threshold for YFP-rhTRIM5α, and all YFP-rhTRIM5α bodies over a volume of 0.011 μm^3^ were used in the analysis. Deconvolved images were analyzed for SUMO-1 maximum fluorescence intensity (MFI) in cytoplasmic bodies using the Surface Finder function of the Imaris software package (Bitplane) and the data was plotted in Prism (Graphpad Software Inc) for statistical analysis.

### Dual-luciferase reporter assay

#### SIMs

293T cells seeded in a 96-well plate were transfected with empty vector, ΔRING/SPRY rhTRIM5α (ΔRS, negative control), wild type rhTRIM5α, SIM1 mut, SIM2 mut, SIM3 mut or RIG-1 (positive control) in triplicate. Transfection was carried out using polyethylenimine (PEI) protocol in which the constructs were added at a 9 (EV/rhTRIM5α/RIG-I): 3 (NF-kB-responsive firefly luciferase construct): 1 (Renilla luciferase construct for transfection efficiencies) ratio. Cells were lysed 48-hours post transfection with Passive lysis buffer (Promega) and the luciferase activity was measured using a Dual-Glo luciferase assay system (Promega) in a Veritas Microplate luminometer. Firefly luciferase data were normalized to Renilla luciferase readings in each well. Data were plotted by determining the fold increase over empty vector.

#### SUMO-1 overexpression

293T cells seeded in a 96-well plate were transfected with empty vector, and rhTRIM5α constructs in presence or absence of SUMO-1 in triplicate. Transfection was carried out using PEI protocol in which the constructs were added at a 5 (rhTRIM5α): 4 (SUMO-1 or EV): 3 (NF-κB-responsive firefly luciferase construct): 1 (Renilla luciferase construct for normalization of transfection efficiencies) ratio. Cells were lysed 48-hours post transfection with Passive lysis buffer (Promega) and the luciferase activity was measured using a Dual-Glo luciferase assay system (Promega) in a Veritas Microplate luminometer. Firefly luciferase data were normalized to Renilla luciferase readings in each well. Data were plotted by dividing SUMO-1 siRNA NF-κB activation by Control siRNA NF-κB activation x 100.

#### SUMO-1 knockdown

293T cells seeded in a 12-well plate were transfected with Control siRNA or SUMO-1 siRNA (Santa Cruz Biotechnology, Inc) following a Lipofectamine2000 (Invitrogen) protocol for two days. On the third day the cells were seeded in a 96-well plate in triplicate and transfected with empty vector or wild type rhTRIM5α using PEI in which constructs were added at a 9 (EV/rhTRIM5α): 3 (NF-kB-responsive firefly luciferase construct): 1 (Renilla luciferase construct for transfection efficiencies) ratio. Cells were lysed 48-hours post transfection with Passive lysis buffer (Promega) and the luciferase activity was measured using a Dual-Glo luciferase assay system (Promega) in a Veritas Microplate luminometer. Firefly luciferase data were normalized to Renilla luciferase readings in each well. Data were plotted by dividing SUMO-1 siRNA NF-κB activation by Control siRNA NF-κB activation x 100.

## Abbreviation

SUMO: Small ubiquitin-related protein; SIM: SUMO-interacting motif; rhTRIM5α: Rhesus monkey TRIM5α; HIV-1: Human immunodeficiency virus; KD: Knocked down.

## Competing interests

The authors do not have competing interests.

## Authors’ contributions

GA designed the research and performed experiments. ZL designed and performed experiments. EMC designed experiments and analyzed the data. SPG discussed and analyzed the data. GA and EMC wrote the manuscript, and all authors read the manuscript and added their corrections. All authors read and approved the final manuscript.
